# Novel likely pathogenic variant in the *EYA1 gene* causing Branchio oto renal syndrome and the exploration of pathogenic mechanisms

**DOI:** 10.1186/s12920-024-01858-y

**Published:** 2024-04-16

**Authors:** Hui Zhang, Jian Gao, Hanjun Wang, Mengli Liu, Shuangshuang Lu, Hongen Xu, Wenxue Tang, Guoxi Zheng

**Affiliations:** 1 Department of Otorhinolaryngology Head and Neck Surgery, the Second Affiliated Hospital of Xi’an Jiaotong University, 710000 Xi’an, China; 2 Department of Otorhinolaryngology Head and Neck Surgery, the Second Affiliated Hospital of Zhengzhou University, 450014 Zhengzhou, China; 3 National Center for International Research in Cell and Gene Therapy, Sino-British Research Centre for Molecular Oncology, School of Basic Medical Sciences, Academy of Medical Sciences, Zhengzhou University, 450052 Zhengzhou, China; 4 BGI College and Henan Institute of Medical and Pharmaceutical Sciences, Zhengzhou University, 450052 Zhengzhou, China; 5 Precision Medicine Center, Academy of Medical Science, Zhengzhou University, 450052 Zhengzhou, China

**Keywords:** *EYA1* gene, Branchio-oto-renal syndrome, Whole-exome sequencing

## Abstract

**Objective:**

Branchio-oto-renal syndrome (BOR, OMIM#113,650) is a rare autosomal dominant disorder that presents with a variety of symptoms, including hearing loss (sensorineural, conductive, or mixed), structural abnormalities affecting the outer, middle, and inner ear, branchial fistulas or cysts, as well as renal abnormalities.This study aims to identify the pathogenic variants by performing genetic testing on a family with Branchio-oto-renal /Branchio-otic (BO, OMIM#602,588) syndrome using whole-exome sequencing, and to explore possible pathogenic mechanisms.

**Methods:**

The family spans 4 generations and consists of 9 individuals, including 4 affected by the BOR/BO syndrome. Phenotypic information, including ear malformation and branchial cleft, was collected from family members. Audiological, temporal bone imaging, and renal ultrasound examinations were also performed. Whole-exome sequencing was conducted to identify candidate pathogenic variants and explore the underlying molecular etiology of BOR/BO syndrome by minigene experiments.

**Results:**

Intra-familial variability was observed in the clinical phenotypes of BOR/BO syndrome in this family. The severity and nature of hearing loss varied in family members, with mixed or sensorineural hearing loss. The proband, in particular, had profound sensorineural hearing loss on the left and moderate conductive hearing loss on the right. Additionally, the proband exhibited developmental delay, and her mother experienced renal failure during pregnancy and terminated the pregnancy prematurely. Genetic testing revealed a novel heterozygous variant NM_000503.6: c.639 + 3 A > C in the *EYA1* gene in affected family members. In vitro minigene experiments demonstrated its effect on splicing. According to the American College of Medical Genetics (ACMG) guidelines, this variant was classified as likely pathogenic.

**Conclusion:**

This study highlights the phenotypic heterogeneity within the same family, reports the occurrence of renal failure and adverse pregnancy outcomes in a female patient at reproductive age with BOR syndrome, and enriches the mutational spectrum of pathogenic variants in the *EYA1* gene.

**Supplementary Information:**

The online version contains supplementary material available at 10.1186/s12920-024-01858-y.

## Introduction

Branchio-oto-renal syndrome (BOR, OMIM#113,650) is a rare autosomal dominant disorder that presents with a variety of symptoms, including hearing loss (sensorineural, conductive, or mixed), structural abnormalities affecting the outer, middle, and inner ear, branchial fistulas or cysts, as well as renal abnormalities [1]. When renal dysplasia is not present, BOR syndrome is sometimes referred to as branchio-oto (BO, OMIM#602,588) syndrome, which shares clinical symptoms and genetic factors similar to BOR. The prevalence of BOR/BO syndrome in the population is approximately 1 in 40,000, affecting about 2% of children with severe hearing loss [2]. The clinical phenotype of BOR/BO syndrome exhibits considerable heterogeneity among individuals and even within the same family [3]. While diagnosing BOR/BO syndrome primarily relies on clinical symptoms, identifying pathogenic variants is essential for understanding the pathogenic mechanism of BOR/BO syndrome in affected families. The known disease-causing genes for BOR/BO syndrome are *EYA1* and *SIX1* [4, 5]. The *EYA1* gene, located on chromosome 8q13.3, which encodes a transcriptional coactivator and comprises 18 exons spanning a length of 159 kb. The EYA1 protein consists of 592 amino acids, featuring a transactivation domain at the amino terminus and an EYA domain (ED) at the C-terminus. Eya1 has no apparent DNA-binding ability and is translocated from the cytoplasm to the nucleus by Six and serves as a co-activator of Six in the regulation of downstream genes [6, 7]. The *SIX1* gene, also known as sine oculis homeobox homolog, encodes a transcription factor (SIX1) that functions as a DNA-binding protein in combination with EYA1 [3]. A recent study also indicates that EYA1 might exert repression activities by interacting with various cofactors [8]. Pathogenic variants in the *EYA1* gene are responsible for the majority (approximately 40%) of BOR/BO syndrome cases [5, 9]. Known *EYA1* variants mainly are loss-of-function, which typically imply haploinsufficiency [10]. To date, more than 200 reported pathogenic variants in the *EYA1* gene are associated with BOR/BO syndrome globally. Limited research publications are available on BOR/BO syndrome in China. Only slightly over 10 pathogenic variants in the *EYA1* gene have been identified in Chinese thus far [11–17]. In this study, we employed whole-exome sequencing to analyze a family affected by BOR/BO syndrome to identify the pathogenic variants. We discovered a novel pathogenic variant in the *EYA1* gene and explored its pathogenic mechanism and clinical features.

## Materials and methods

### Study subjects and clinical evaluation

This study investigates a hearing-loss family that sought medical care at the Second Affiliated Hospital of Zhengzhou University in February 2022. The family consists of four generations and includes nine individuals, four of whom are affected by the disease. The clinical assessments conducted in this study encompass otoscopy, pure-tone audiometry, tympanometry, distortion product otoacoustic emission (DPOAE), auditory brainstem response (ABR), multiple-frequency auditory steady-state evoked response (ASSR), and 40 Hz auditory event-related potential (40 Hz AERP) tests. Temporal bone CT, renal function, and renal ultrasound evaluations are also included. The clinical diagnosis of BOR/BO syndrome is based on the criteria proposed by Chang et al. in 2004. To receive a clinical diagnosis, a patient must meet any of the following criteria: the presence of three or more major symptoms; the presence of two major symptoms and two minor symptoms; the presence of one major symptom and a family member with BOR/BO syndrome [9]. Hearing impairment was classified according to the World Health Organization classification of hearing loss in 2021 as follows: mild (20–35 dB), moderate (35–50 dB), moderately severe (50–65 dB), severe (65–80 dB), profound (80–95 dB), and complete hearing loss (≥ 95 dB).

This study follows the ethical guidelines of the Helsinki Declaration, and the research protocol has received approval from the Ethics Committee of The Second Affiliated Hospital of Zhengzhou University (Approval Number: 2,018,008). Written informed consent was obtained from all participating individuals or their guardians prior to enrollment in the study.

### Whole-exome sequencing

A total of 3 mL of peripheral venous blood was collected from the affected individuals for whole-exome sequencing. Genomic DNA was extracted using the GenMagBio Genomic DNA Purification kit (GenMagBio, Changzhou, China) according to the manufacturer’s protocol. Following fragmentation and end-repair of the genomic DNA from the proband, adapter ligation, and PCR enrichment were conducted as per the manufacturer’s instructions for the VAHTS TM Universal DNA Library Prep Kit for Illumina V3 (Vazyme Biotech Co., Ltd, Nanjing, China). The SureSelect Human All Exon V7 (Agilent) was utilized to capture the exons and the flanking regions of all known genes. The library was subsequently sequenced using an Illumina HiSeq 4000 sequencer in pair-end 150 mode at the Precision Medicine Center of Zhengzhou University, Zhengzhou, China [18].

### Bioinformatics analysis, variant interpretation, and sanger sequencing

Bioinformatics analysis was performed in the framework of bcbio-nextgen (https://github.com/bcbio/bcbio-nextgen), which provides best-practice pipelines for variant calling, annotation, and validation [18]. After sequencing adaptors and inferior reads had been removed from raw data, clean reads were mapped to the human reference genome (version GRCh37) using Burrows-Wheeler Aligner (version 0.7.17-r1188). Duplicate reads were flagged by Sambamba (version 0.6.6) [19]. Single-nucleotide variations and small insertions or deletions were investigated with the Genome Analysis Toolkit HaplotypeCaller [20]. Variant annotation, filtering, and interpretation were performed as described previously [17, 18]. Polymerase chain reaction (PCR) amplification and Sanger sequencing were performed to validate candidate variants detected by whole-exome sequencing. Amplified PCR products were purified by a PCR purification kit (LifeSciences, Hangzhou, China) and then sequenced using the SeqStudio Genetic Analyzer (Applied Biosystems/Life Technologies, Carlsbad, CA, United States). Variant nomenclature was based on *EYA1* canonical transcript NM_000503.6.

### Minigene experiments

To construct plasmids for minigene experiments, we utilized the proband DNA as a template for PCR amplification. Two pairs of Nested PCR primers, namely EYA1-F and EYA1-R, as well as MINI-EYA1-Not-R and MINI-EYA1-Kpn1-F, were employed for the Nested PCR process. Subsequently, the second round of PCR products underwent PCR with Exon8-Kpn-R and Exon8-Not-F primers. This allowed us to obtain both wildtype and mutant fragments comprising intron 7, exon 8, and intron 8. These fragments were then utilized for the construction of plasmids intended for the minigene experiment.

For the minigene experiment, we utilized the plasmids described above to investigate the impact of the candidate variant in exon 8 on mRNA splicing. These plasmids contained intron 7 (501 bp), exon 8 (83 bp), intron 8 (264 bp), and universal sequences (exon A-intron A… intron B-exon B). Both wildtype and mutant plasmids were transfected into HEK293 cells and HeLa cells transiently. After 48 h, we collected total RNA and performed reverse transcription. Subsequently, we conducted PCR using cDNA as the template and PCMINI-F and PCMINI-R primers (Table 1). The size of the PCR product was determined through 1% agarose gel electrophoresis. The gel images revealed distinct band sizes for the wild type and mutant type. We recovered the target bands and verified them through Sanger sequencing, performed by Sangon Biotech (Shanghai) Co., Ltd.

## Results

### Clinical characteristics

**Fig. 1:**
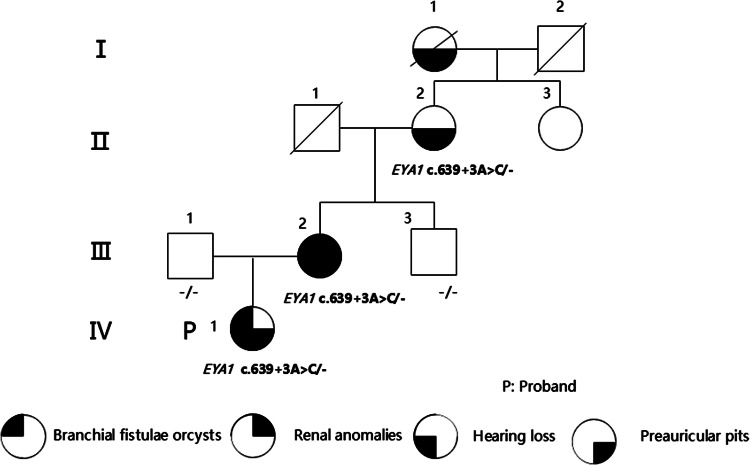
Pedigrees of the family with BOR/BO syndrome. The family spans four generations, with 4 individuals affected with different combinations of phenotypes, as indicated in the figure. Circles denote females; squares denote males; different black parts represent differential phenotype

The family consists of 9 individuals spanning four generations, with four individuals affected by the syndrome. The parents of the proband were not closely related. There were no instances of infection or exposure to toxic substances during pregnancy. Neither the proband nor other affected family members exhibited tinnitus or vertigo, and there was no history of noise exposure, ototoxic drug usage, or external ear trauma. After collecting relevant clinical information from family members, a pedigree chart was drawn (Fig. 1).

**Fig. 2:**
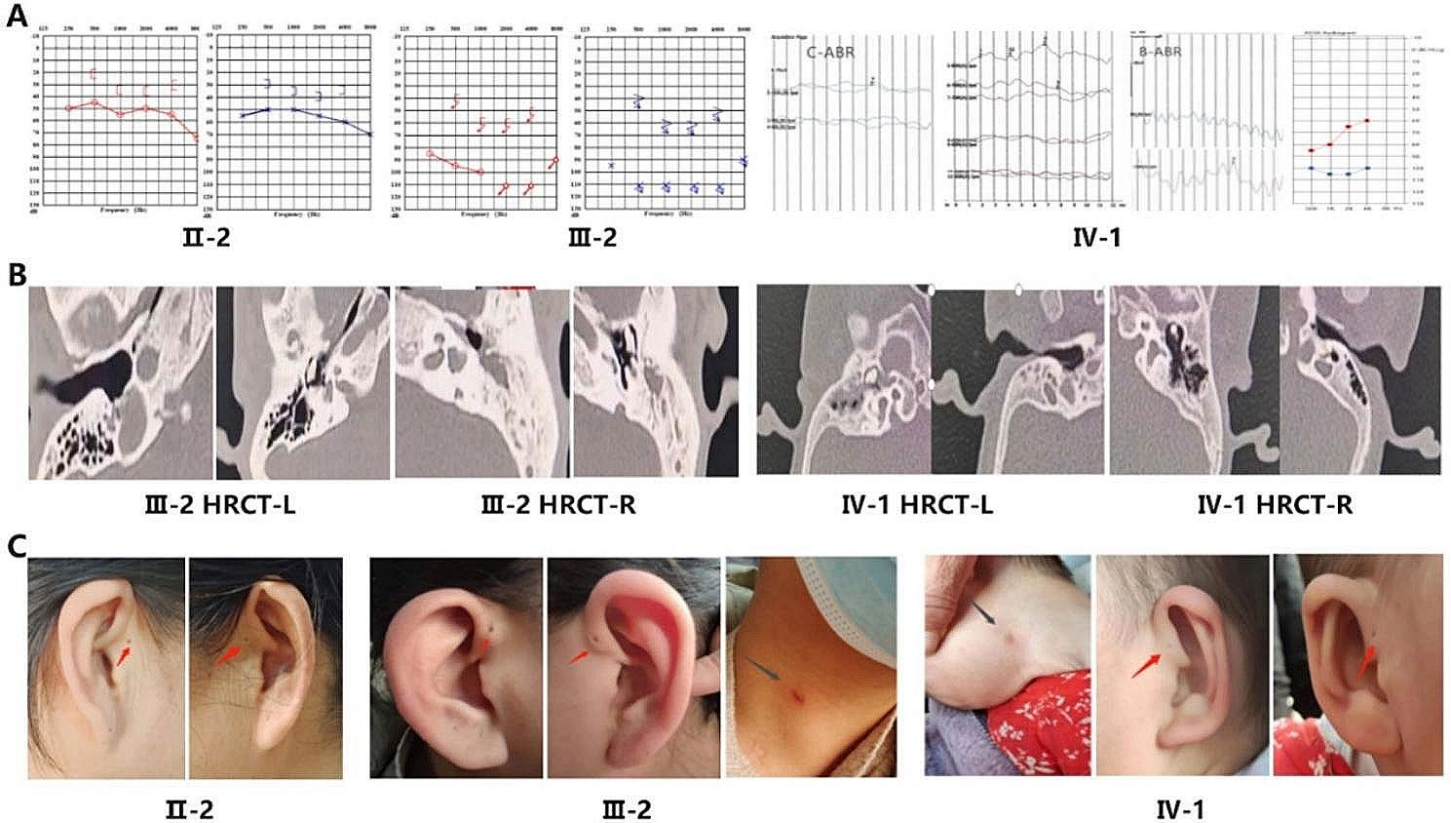
Audiograms, CT images of the temporal bone and clinical features. (**A**) pure-tone audiometry, auditory brainstem response or auditory steady-state response; (**B**) Axial CT images of the temporal bone from subject III-2 and IV-1; (**C**) medical photographs of patients: Black arrow: branchial cleft fistulae or scar; red arrow: preauricular pits

The proband in this family is IV-1, a 1-year and 8-month-old girl who did not pass the universal newborn hearing screening. She was born prematurely at 31 + 6 weeks gestation, weighing 1.13 kg. Her Apgar scores were 7 at 1 min (with muscle tone, breathing, and reflex all scoring − 1) and 9 at 5 and 10 min (with breathing scoring − 1 at 5 min). She received neonatal intensive care unit (NICU) treatment for 52 days and was diagnosed with several conditions, including mild neonatal asphyxia, prematurity, extremely low birth weight, neonatal respiratory distress syndrome, neonatal hyperbilirubinemia, necrotizing enterocolitis in neonates, neonatal sepsis, premature infant brain injury, and premature infant anemia. The patient is now seeking medical attention because of language difficulties and associated growth retardation. Physical examination revealed short stature, bilateral auricular deformities (drooping ears), a preauricular sinus, oily cerumen in both ears, a clear external auditory canal and visible tympanic membrane in the right ear, and a narrow external auditory canal with an unobservable tympanic membrane in the left ear. Additionally, a visible sinus tract was observed in the left neck. Audiological examination revealed profound sensorineural hearing loss in the left ear and moderate mixed hearing loss in the right ear. CT scan on the temporal bone showed a narrow left external auditory canal, malformations in both the middle and inner ear, abnormal development of the malleus and incus, a cystic cochlea, vestibular malformation, and a large cystic vestibule without cochlear axis (Fig. 2). A color Doppler on the renal showed no abnormalities. The proband received rehabilitation treatment at a local hospital, which improved language and motor skills.

Patient III-2, the mother of the proband, is deaf and mute and has not received formal medical treatment. During her pregnancy, she delivered a premature female infant (IV-1) due to preeclampsia. She currently has end-stage renal disease (ESRD) and is undergoing dialysis treatment. Physical examination reveals normal morphology of both auricles, bilateral preauricular sinuses, and clear external ear canals with oily cerumen. The tympanic membrane is visible, and a branchial cleft cyst is observed in the right neck. Audiological examination shows severe bilateral sensorineural hearing loss. High-resolution computed tomography (CT) on the temporal bone reveals ossicular malformation, cystic cochlea, and an enlarged vestibular aqueduct (Fig. 2). Color Doppler imaging on the renal and ureteral shows small kidney size, with the left and right kidney sections measuring 72 × 46 × 43 mm and 81 × 60 × 40 mm, respectively. The right kidney appears well-rounded with diffuse enhancement of parenchymal echoes, and there is indistinct corticomedullary differentiation. Both the renal pelvis and calyces are dilated.

Patient II-2 is the grandmother of the proband and has been experiencing hearing loss since childhood, but is still able to communicate in daily life. Physical examination reveals normal morphology of both auricles, bilateral preauricular sinuses, and clear external ear canals with oily cerumen. The tympanic membranes are intact and no branchial cysts are observed in the neck. Audiological examination shows bilateral moderate mixed hearing loss (Fig. 2). CT scan on temporal bone and color Doppler on renal were not performed.

### Genetic testing reveals a novel *EYA1* variant

**Fig. 3:**
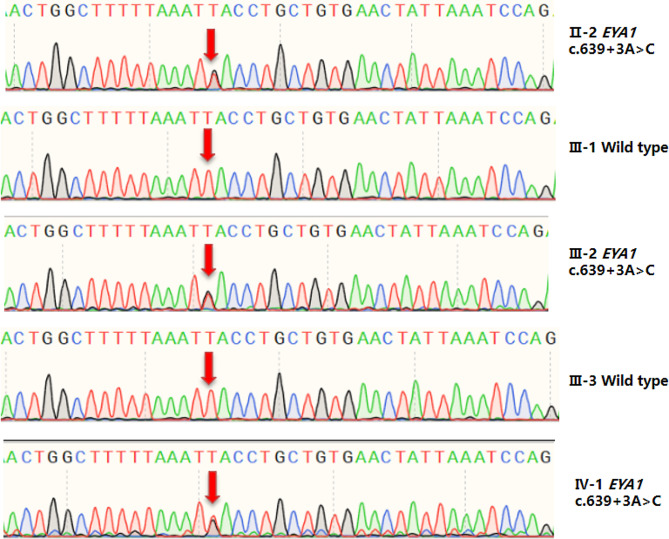
Sanger sequencing of the *EYA1* variant c.639 + 3 A > C in the family. The Sanger sequencing of the variant site c.639 + 3 A > C in the family members, with red arrows indicating the variant sites. Please note that *EYA1* is in reverse strand, so the variants in the figure was shown as T > G

Whole exome sequencing (WES) was performed on proband, and variants in BOR syndrome genes (*EYA1* and *SIX1*) and other genes were evaluated to associate with hearing loss (definitive, strong, and moderate) by ClinGen Hearing Loss Clinical Domain Working Group were filtered [21].Heterozygous variants *OTOG* (NM_001292063.2):c.2402 A > G and *EYA1* (NM_000503.6):c.639 + 3 A > C (ClinVar accession ID: SCV004176794) were identified. The former one is not further considered as homozygous or compound heterozygous variants in the *OTOG* gene cause autosomal recessive non-syndromic deafness-18B. The *EYA1* variant c.639 + 3 A > C has not been reported in the gnomAD database (PM2_Supporting) or in the literature. The variant co-segregates with the phenotype in the family (PP1, Fig. 3). The phenotype associated with the *EYA1* gene is consistent with the phenotype observed in this family (PP4). dbscSNV and SpliceAI predict that this variant may affect splicing (dbscSNV ada score: 1, dbscSNV RF score: 0.98, spliceAI donor loss: 0.83, PP3). Therefore, based on ACMG guidelines, this variant was initially classified as a variant of uncertain significance (VUS).

### Minigene assay shows that the variant affects splicing

To further investigate the potential impact of this variant on splicing, a minigene assay was conducted. The minigene was constructed by inserting the sequence of exon 8 and its adjacent intronic regions into the pcMINI vector, which contains the universal ExonA-IntronA-IntronB-ExonB sequences. Following transfection into cells, any abnormalities in the splicing pattern of ExonA-Exon8-ExonB were observed.

**Fig. 4:**
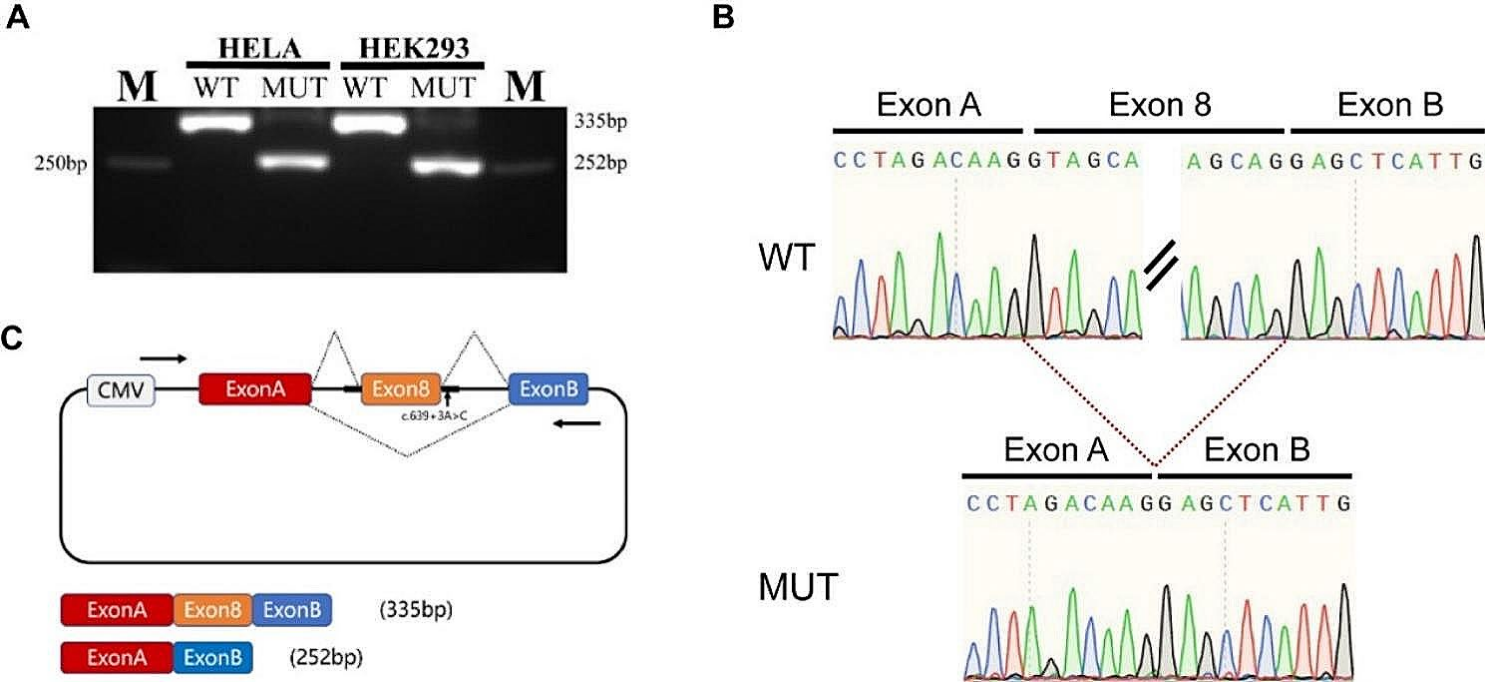
The effect of the variant c.639 + 3 A > C on splicing by minigene assay. The gel electrophoresis results of PCR products using cDNA as a template with PCMINI-F and PCMINI-R primers show a band of 335 bp in the WT and two bands of 335 bp and 252 bp in the MUT with the larger weak band corresponding to the one in WT; The ratio of band a (larger band) to band b (smaller band) in MUT is 1:9. (**B**) Sanger sequencing results of 335 bp and 252 bp PCR products showed two different splicing patterns; (**C**) Schematic representation of minigene assay design and transcript products in WT and MUT

RT-PCR analysis was performed, and the mutated sample from HeLa and HEK293 cells showed two bands. The larger band, labeled as “a” corresponded to the expected size and represented the normal spliced transcript in wild-type. The smaller band, labeled as “b” was only present in the mutated sample. Both bands were sequenced, and the sequencing results indicated that band “a” represented the normal spliced transcript with the ExonA-Exon8-ExonB splicing pattern. Band “b” showed a complete absence of exon 8, indicating a splicing pattern of ExonA-ExonB.The ratio of band a to band b in MUT is 1:9. (Fig. 4). These results showed that the variant c.639 + 3 A > C may cause the skipping of exon 8 in this family. We deduced the resulting amino acids and found that the transcripts lacking exon 8 extended 17 amino acids before the creation of premature stop codon. The resulting abnormal transcript may be degraded by nonsense-mediated mRNA decay, leading to haploinsufficiency. Therefore, based on ACMG guidelines, this variant is reclassified as likely pathogenic (PM2_supporting, PP1, PP4, and PS3).

## Discussion

BOR/BO syndrome exhibits considerable clinical heterogeneity, which increases the risk of misdiagnosis and underdiagnosis in clinical settings. Early identification becomes particularly challenging when the hearing loss is mild, the branchial fistula/cysts are absent, and there are no accompanying renal abnormalities. The widespread use of genetic testing for genes associated with hearing loss has made early diagnosis of BOR/BO syndrome feasible in clinical practice. In this study, we identified a novel pathogenic variant c.639 + 3 A > C in the *EYA1* through WES and revealed its effect on splicing by minigene assay. The establishment of a causal relationship between the pathogenic variant and the disease has promoted clinical management and genetic counseling for patients in this family.

In the present study, all patients meet the clinical diagnostic criteria [9]. There is a significant variation in renal and hearing phenotypes within this family, not only among different members but also within the same patient. The presence of interfamilial phenotypic variability has been confirmed, while the phenotypic differences on the left and right sides of a patient are relatively rare. Research has found that interventions for hearing loss in BOR/BO syndrome have limited effectiveness [22]. In this study, the proband initially presented with serous otitis media in the right ear. The tympanogram showed a type B curve. After receiving medical treatment, the middle ear effusion resolved, and the tympanogram showed a type A curve. However, the hearing did not significantly improve, possibly indicating a conductive hearing loss in the right ear. After the resolution of otitis media, there was no significant improvement in hearing.

In addition to the typical manifestations of the ear, gill, and kidney abnormalities, BOR/BO syndrome may also be associated with other symptoms, including developmental delay, intellectual disability, hypospadias, skeletal defects, and feeding difficulties [23, 24]. When diagnosing and treating BOR/BO syndrome, the overall systemic manifestations should be paid attention. The proband IV-1 exhibited symptoms of anemia, feeding difficulties, and respiratory distress during the neonatal period. She showed growth retardation, with below-average height, weight, cognition, motor skills, and language abilities compared to her peers now. Previously, it was believed that the accompanying symptoms were directly or indirectly caused by the clinical manifestations of gill, ear, and kidney abnormalities. For example, Renal dysfunction can lead to growth retardation and intellectual disability, while inadequate neck development can cause feeding difficulties [25, 26]. An infant with BOR syndrome presenting with poor growth and hypoglycemia was reported due to growth hormone deficiency [27]. However, the IV-1 was born extremely premature, And the growth retardation may be attributed to prematurity and neonatal encephalopathy. Further research is needed to determine if the pathogenic variants in genes related to BOR/BO syndrome can cause other accompanying symptoms.

To the best of our knowledge, patient III-2 in this family is the first reported case of BOR syndrome worldwide, whose renal failure was due to her failure to seek medical attention. Reports indicate that approximately 66% of BOR/BO syndrome patients have coexisting renal developmental abnormalities, and 6% of them progress to renal failure [28]. Severe kidney disease can lead to chronic renal failure within 20 years of birth [28, 29]. In China, the prevalence of renal phenotypes is relatively low, typically presenting as BOS [17, 30]. The age of onset for end-stage renal disease (ESRD) in patients with renal phenotypes remains unclear, and the existence of triggering factors has not been reported. However, it is evident that pregnancy can have detrimental effects on renal disease in women with preexisting kidney conditions [31, 32]. BOR syndrome is a rare clinical condition, and there have been no reports on the perinatal outcomes of women of reproductive age with BOR syndrome and associated renal developmental abnormalities. The system evaluation revealed that patients with chronic kidney disease have a 5-fold increased risk for mothers and a 2-fold increased risk for fetuses compared to normal pregnancies [31]. However, among women with underlying glomerular diseases, if their condition is stable and kidney function is preserved, the occurrence rate of adverse events during pregnancy is lower [32]. Therefore, it is essential to strengthen interdisciplinary collaboration to enhance understanding of BOR syndrome and to promptly identify, diagnose, and intervene in renal abnormalities.

The variant c.639 + 3 A > C in the *EYA1* gene is identified as the pathogenic variant in this family. *EYA1* plays a crucial role as a transcription factor in the kidney and the development of the first and second branchial arches [33]. The *SIX1* transcription factor is essential for regulating transcription in the nucleus and *EYA1* acts as a cofactor and binds to *SIX1*, forming a bipartite transcription factor [34, 35]. EYA1 functions as a transcription co-activator and interacts with SIX1, thus providing a molecular mechanism of BOR/BO syndrome. In the absence of this interaction, the transcriptional activation of downstream targets required for the development of the branchial, otic, and renal systems is diminished [36]. Functional studies have confirmed that genetically defective *EYA1* and *SIX1* mice exhibit symptoms similar to BOR/BO syndrome [37]. In this study, we showed that c.639 + 3 A > C caused the skipping of exon 8 in minigene, which may lead to the creation of premature stop codons, and trigger the degradation of the transcript by the nonsense-mediated decay pathway. RT-PCR analysis of *EYA1* transcript in fibroblasts from a patient of BOR heterozygous for NM_172060.2:c.867 + 5G > A (NM_000503.6:c.966 + 5G > A) identified a truncated transcript of *EYA1* lacking exon 8 (NM_172060.2), predicting a frameshift and premature termination signal [38]. But based on NM_172060.2, the variant c.867 + 5G > A will be c.966 + 5G > A, which will result in exon 10 skipping. In addition, c.639 + 1G > A and c.639 + 1G > C may have the same effect as c.639 + 3 A > C, but there were no functional data available [39, 40]. The limitation of the study is that we cannot confirm the aberrant transcript in vivo due to sample unavailability. The relationship between different mutation types and the clinical phenotype of BOR/BO syndrome is still not fully understood, and the diverse and complex clinical phenotype observed in this family further emphasizes this point [41, 42].

In conclusion, pathogenic variants in the *EYA1* gene can result in a wide range of symptoms and severity in BOR/BO syndrome. The most significant factor impacting the quality of life for patients, particularly reproductive-age females with BOR/BO syndrome, is kidney disease. Therefore, it is crucial to diagnose kidney abnormalities early, address their underlying causes, and prevent the progression to ESRD. Additionally, it is essential to enhance pregnancy management for reproductive-age females in BOR/BO syndrome families, provide genetic counseling for early diagnosis, conduct genetic screening and prenatal diagnosis, such as amniocentesis, during pregnancy, and correct developmental abnormalities in newborns, including external and middle ear defects, through surgical intervention. These measures aim to achieve effective prevention and treatment strategies.

**Table 1:** Primer sequences used in minigene assay

Primer name	Primer sequence (5’- 3’)
*EYA1*-F	GCTCTGGAAACAGGCAAGAGG
*EYA1*-R	TGCAGTGTATGAATGCCGTTT
MINI-*EYA1*-BamH1-R	tagtGGATCCGGTGCTGCCATTCATTCTT
MINI-*EYA1*-Kpn1-F	ggtaGGTACCGCTCTATATGTGGTGCCAA
Exon8-Kpn-R	TAGTGGTACCCGGTGCTGCCATTCATTCTT
Exon8-Not-F	GGTAGCGGCCGCGAGCTCAGACTTGCAAAC
PcMINI-F	ctagagaacccactgcttac
PcMINI-R	tagaaggcacagtcgagg

### Electronic supplementary material

Below is the link to the electronic supplementary material.

Supplementary Material 1

## Data Availability

The EYA1 varint found in this study was submitted to the ClinVar repository [https://www.ncbi.nlm.nih.gov/clinvar/variation/2671884/].
