# Supplementary figures and images for: Novel likely pathogenic variant in the EYA1 gene causing Branchio oto renal syndrome and the exploration of pathogenic mechanisms
Source: BMC Med Genomics. 2024 Apr 16;17:89. doi: 10.1186/s12920-024-01858-y (PMC11020176; doi:10.1186/s12920-024-01858-y)

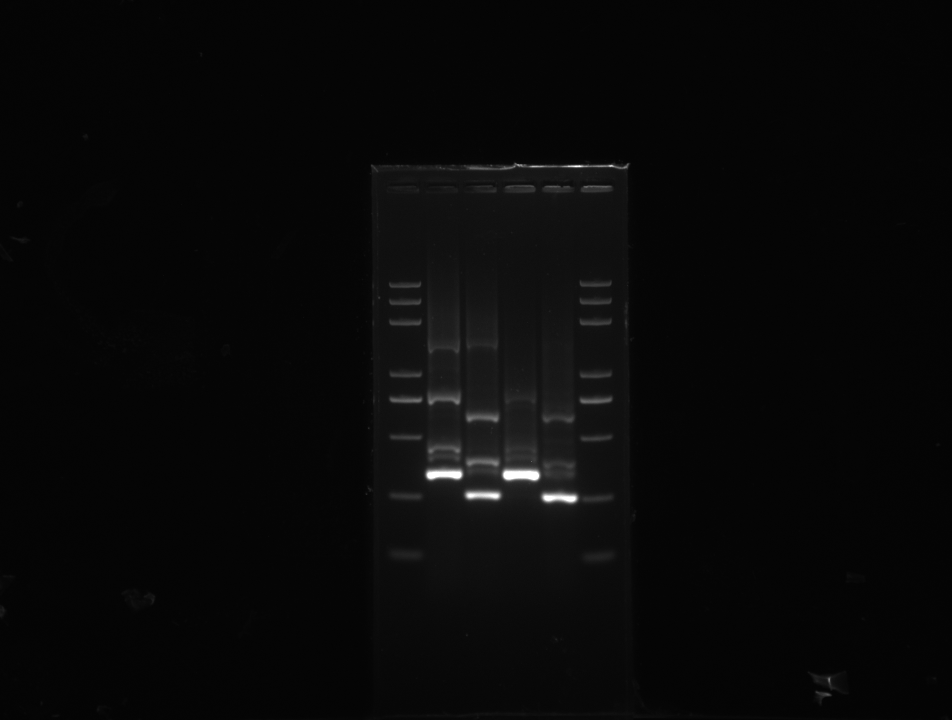


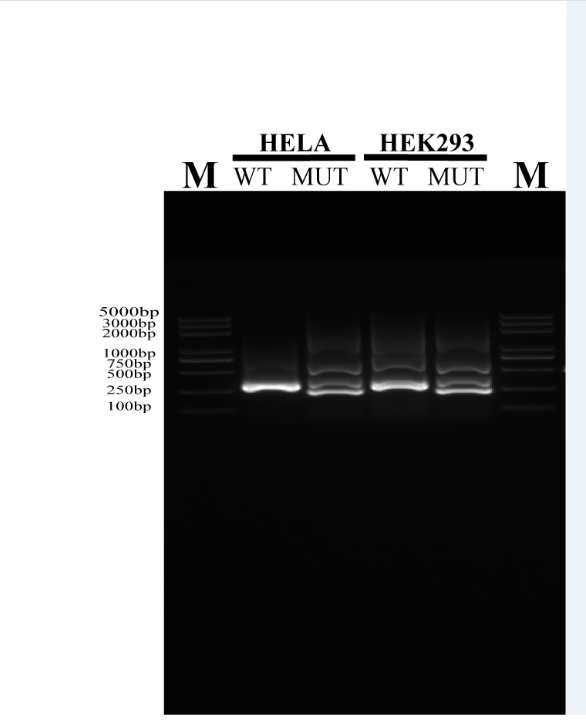


Exon8-full-1(1)


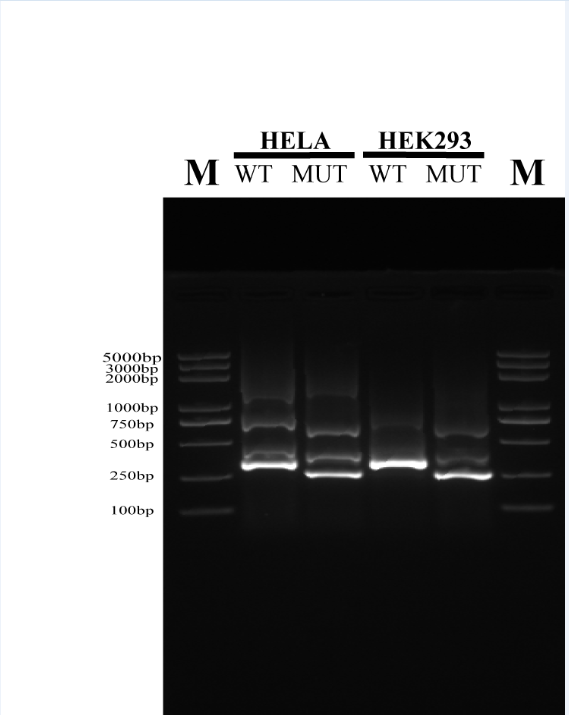


Exon8-full-2(1)


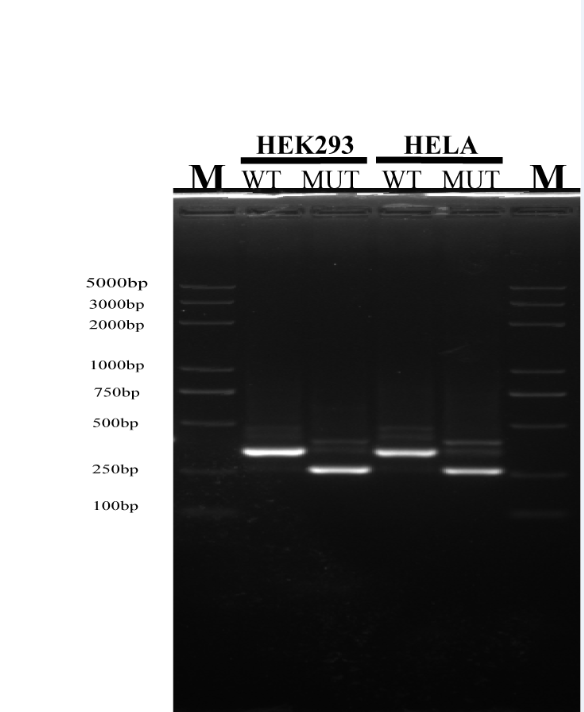

Supplement: Supplementary file 1 — Supplementary Material 1 [file 12920_2024_1858_MOESM1_ESM.docx]
